# A ceramic bone substitute containing gentamicin gives good outcome in trochanteric hip fractures treated with dynamic hip screw and in revision of total hip arthroplasty: a case series

**DOI:** 10.1186/s12891-018-2360-8

**Published:** 2018-12-06

**Authors:** Mindaugas Stravinskas, Sarunas Tarasevicius, Simonas Laukaitis, Malin Nilsson, Deepak Bushan Raina, Lars Lidgren

**Affiliations:** 1 Lithuanian University of Health, Kaunas, Lithuania; 2 Department of Orthopedics, Lund University Hospital, Lund, Sweden

**Keywords:** Hip surgery, Infection, Ceramic bone substitute, Antibiotics, Gentamicin, Bone remodeling

## Abstract

**Abstract:**

**Background:**

The primary objective was to investigate the clinical and radiological outcome in patients undergoing major hip surgery using a novel antibiotic containing bone substitute for local augmentation in trochanteric fracture fixation or revision of total hip arthroplasty (THA).

**Methods:**

We implanted a novel biphasic bone substitute CERAMENT™|G consisting of hydroxyapatite, calcium sulphate and gentamicin for bone regeneration and local antibiotic delivery in 20 patients treated surgically for trochanteric femoral fracture or uncemented hip revision. Preoperative, postoperative, 3 months and 1 year clinical and radiological assessment were performed including registration of any complications. In one trochanteric fracture patient, histological analyses were performed of bone biopsies taken at removal of hardware.

**Results:**

None of the trochanteric fractures or revision of THA showed any large migration. No local wound disturbances were seen and no infection was observed at one year follow-up. All trochanteric fractures healed at 3 months with a minimal sliding screw displacement on average 3 mm. Radiological analysis showed signs of bone remodeling and new bone formation in the substitute, illustrated also by histology in the biopsies taken from one trochanteric fracture at one year post-op.

**Conclusions:**

Local CERAMENT™|G was shown to be safe in a limited prospective major hip surgery study. Remodeling of the bone graft substitute was observed in all patients.

**Trial registration:**

EU-CTR2018–004414-18 Retrospectively registered on November 20, 2018.

## Background

Deep bone and joint infections after orthopaedic procedures are a devastating complication which may require revision surgery and longterm systemic antimicrobial treatment [1]. Conventional approaches used to treat acute infections may not work for chronic biofilm infections and in many cases promote antimicrobial resistance and further biofilm formation [2, 3]. Use of antibiotic-impregnated cement spacers is the standard method to provide direct local delivery of antibiotics which are mainly released from the surface of the cement and limited from the voids within the cement. The most extensively studied and commercially available implant for controlled local release of antibiotics is non-biodegradable polymethylmethacrylate (PMMA) bone cement [4].

CERAMENT™|G is an injectable biphasic bone substitute, composed of 60% calcium sulphate, which resorbs and provides gradual porosity for new bone ingrowth, supported by 40% hydroxyapatite that acts as matrix for bone cells. It also contains gentamicin sulphate (175 mg/10 mL). Recent studies have shown promising results of CERAMENT™|G on bone regeneration, and prevention or eradication of infection [5]. Dvorzhinskiy et al. described where CERAMENT™|G was used for treating osteomyelitis in the tibias of rats [6]. The gentamicin containing bone substitute increased new bone growth as compared with empty control in a debrided osteomyelitic environment. Animals treated with CERAMENT™|G showed no evidence of infection and retained a higher bone mass compared to the contralateral (non-operated) side 6 months post-op.

In a previous study, we investigated local elution of gentamicin in patients treated for trochanteric fractures or revision of total hip arthroplasty (THA) when CERAMENT™|G was used for bone defect reconstruction [7]. A high level of gentamicin was initially detected in the wound drainage fluid, followed by a decreasing concentration, while in blood serum gentamicin concentration was below the recommended systemic level. However, we found no studies investigating the clinical and radiological outcomes of trochanteric fractures or revision of THA patients, when bone substitute containing antibiotics was used in major hip surgery. This study is a continuation of our previous work [7] and the aim was to investigate the clinical and radiological outcomes of patients undergoing trochanteric fracture fixation or revision of THA with CERAMENT™|G used for bone defect augmentation.

## Methods

From June 2014 to September 2016, 20 patients with trochanteric fracture internal fixation surgery using a dynamic hip screw (DHS) and aseptic revision of THA with uncemented stem were included in a single center, prospective, observational study. Patients with systemic aminoglycoside usage before surgery, septic process in the hip joint, psychiatric or neurological disorders, renal failure, impaired hearing, and hypersensitivity to aminoglycosides were excluded.

**Fig. 1:**
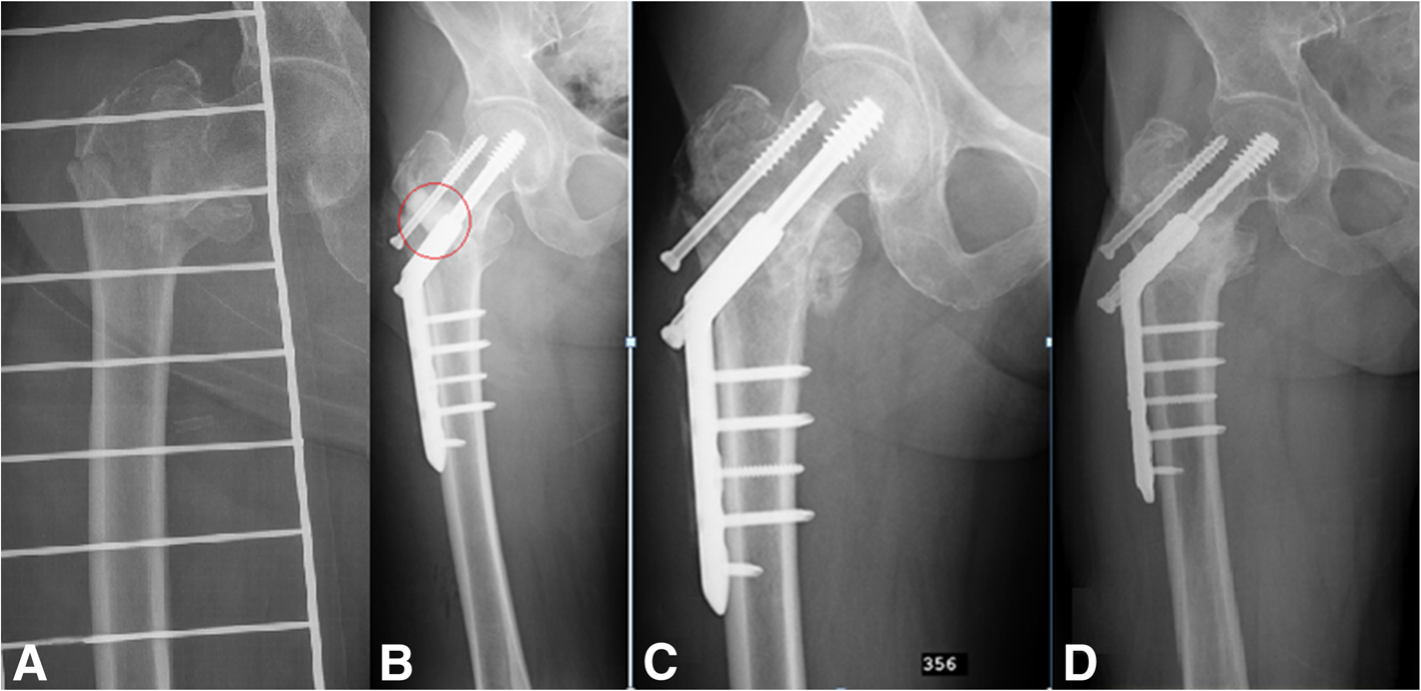
DHS patient‘s proximal part of the femur healing. Preoperative image shows the trochanteric fracture (**a**). In the postoperative image (**b**) CERAMENT™|G is seen (circled in red). In the X-ray image after 3 months (**c**), the bone graft substitute is difficult to differentiate from bone. Image after 1 year (**d**) shows complete healing. This patient needed DHS removal because of local discomfort

**Fig. 2:**
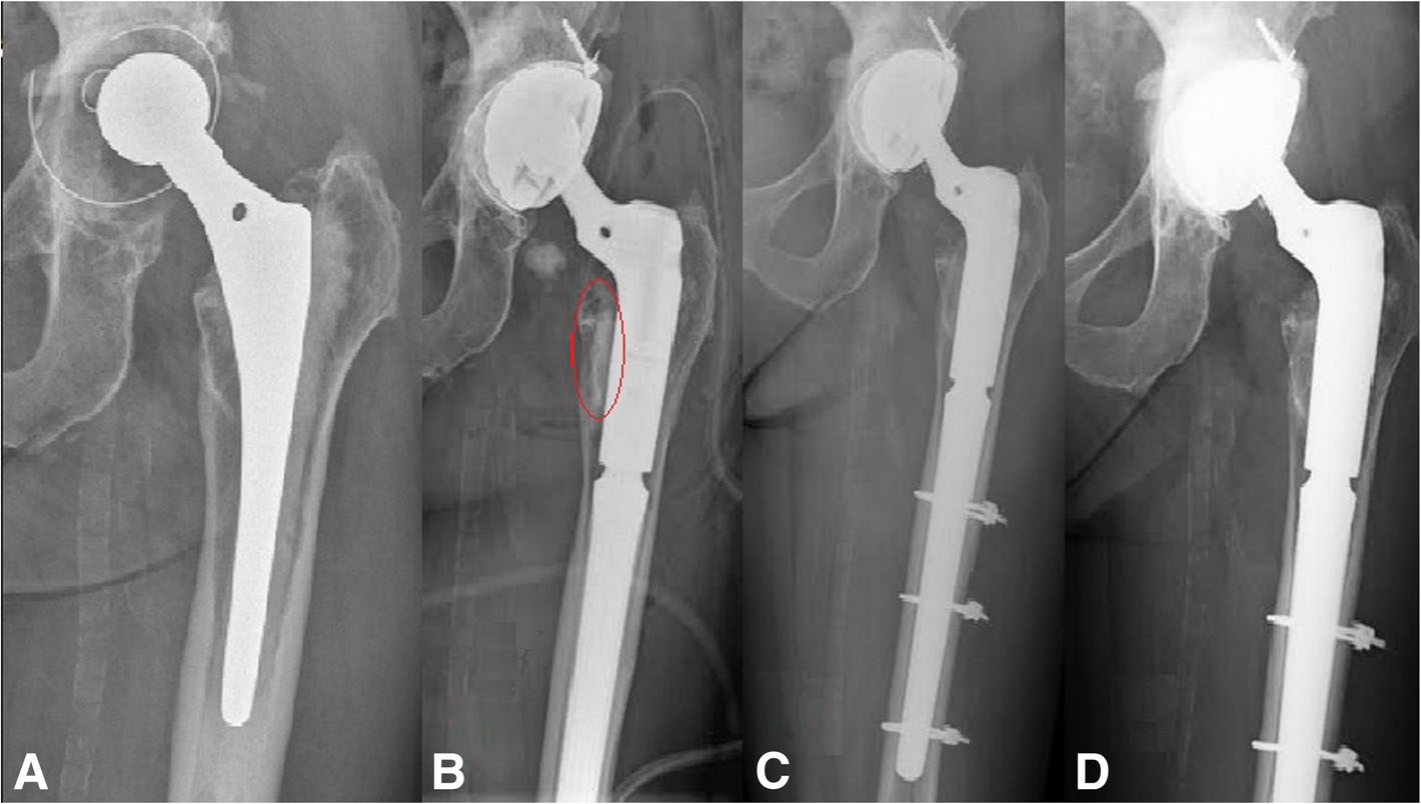
Revision of THA using a cementless femoral component. Preoperative image shows unstable components (**a**). The proximal part of the femur postoperatively (**b**) shows CERAMENT™|G present (circled in red). In the X-ray after 3 months (**c**), the bone graft substitute is difficult to differentiate from bone. Image after 1 year (**d**) shows bone healing

Synthetic bone graft substitute (CERAMENT™|G, BONESUPPORT AB, Lund, Sweden) was used to augment the bone defects at implantation of either DHS or after implantation of cementless femoral component. For the fracture patients, CERAMENT™|G was injected into the bone defect in the trochanteric region after placing the dynamic screw but before plate implantation (Fig. 1). While in the revision of THA cases CERAMENT™|G was injected into visible bone defects around the proximal part of the prosthesis after distal fixation of the uncemented stem (Fig. 2). In cases where a cemented acetabular component was chosen, plain PMMA bone cement (without antibiotic) was used for component fixation.

X-ray examination of the operated site was performed the first day after surgery, and patients were mobilized and started to walk using crutches. Clinical and radiological outcome was performed at 3 months and at 1 year follow-up.

For the trochanteric fracture patients radiological analysis included anteroposterior (AP) and lateral views. We estimated postoperative fracture reposition and it was considered satisfactory if femoral neck angle was < 10° of varus or < 15° of valgus compared to the uninjured contralateral hip and the fracture displacement was < 5 mm on both AP and lateral post-operative radiographs [8].

The position of the lag screw was assessed from the mapping of the femoral head and the tip-apex distance (TAD). Fixation was deemed to be adequate if the lag screw was placed to central/central (AP/lateral view), inferior/central, or inferior/posterior Cleveland zones [9]. A TAD of less than 25 mm was considered optimal. Sliding was measured along the dynamic screw. The fracture was considered as healed if bone trabeculae were visible across the fracture line. Absence of bridging bone at the line of the fracture after 3 months was considered as non-union [10].

For revision of THA patients, the radiological analysis was performed of the proximal part of the femur where the CERAMENT™|G had been injected. Bone density changes such as: new bone formation, cortical healing as well as subsidence of implant were analyzed.

To evaluate functional outcome at 3 months and 1 year follow-up, patients were asked if they had pain at the operated site and if they were able to bear weight on the operated leg. They were also asked to evaluate their quality of life (QoL) from 1 to 100 using VAS (Visual Analog Scale).

Additionally, all wound or systemic complications related to surgery were recorded.

One patient had hardware removal due to local discomfort of the DHS after 1 year. At the time of the surgery a bone biopsy from the CERAMENT™|G implantation site was taken for further histology analysis. The biopsy was fixed in 4% formalin in neutral buffered saline overnight followed by decalcification in 10% EDTA solution for 3 weeks. Routine paraffin embedding procedures were used before sectioning the specimen to a thickness of 5 μm using a semi-automaticmicrotome (Thermo Scientific, U.S.A). Sections were allowed to adhere to coated glass slides for 48 h prior to routine H&E staining.

## Results

The prospective study consisted of 20 patients (3 men, 17 women) with a mean age of 74.8 years, (±SD 9.5 years, range 57–90 years) who underwent either trochanteric fracture fixation using DHS or aseptic revision of THA using uncemented distal fixation modular stem (10 for each group) (Tables 1 and 2). The distal stem used was tapered and the whole stem coated with Porous Plasma Spray (PPS®) (Arcos, ZimmerBiomet, USA). A mean of 7.28 mL (±SD 1.10 mL, range 5–9 mL) of CERAMENT™|G was implanted for patients with trochanteric hip fracture and mean of 7.61 mL (±SD 0.49 mL, range 7–9 mL) for revision of THA patients.

**Table 1:** Trochanteric fracture patient data

Patient No.	Age	Gender	Pain in op leg	Clin function	VAS for QoL 3 months	VAS for QoL 1 year	Outcome	Complications
1	83	F	No	Full	85	90	Healed fr.	–
2	83	F	No	Can‘t walk	30^a^	No foll up	No foll up	–
3	62	F	No	Full	65	88	Healed fr.	–
4	75	F	No	Full	70	80	Healed fr.	–
5	85	F	Died	–	–	–	–	–
8	64	F	No	Difficult	80	80	Healed fr.	–
14	81	F	No	Full	60	80	Healed fr.	–
15	80	F	No	Full	80	90	Healed fr.	–
19	78	F	No	Full	80	80	Healed fr.	–
20	89	F	No	Full	50	90	Healed fr.	–
Mean	78.0				71.3	84.8		
S.D.	8.3				11.4	4.8		

^a^excluded from Mean and S.D. due to no follow-up at 1 year

**Table 2:** Cementless hip revision patient data

Patient No.	Age	Gender	Pain in op leg	Leg function	VAS of QoL at 3 months	VAS of QoL at 1 year	Defect type (Paprovsky classification)		Graft healing	Complications
							acetabulum	femur		
6	67	F	No	Full	60	75	IIb	IIIa	yes	–
7	62	F	No	Full	75	80	IIa	IIIb	yes	–
9	67	M	No	Full	70	70	IIa	IIIb	yes	–
10	90	M	No	Full	90	100	IIIa	IIIa	yes	–
11	84	F	Yes	Full	60	85	IIIa	IIIb	yes	–
12	57	M	No	Full	40	78	IIb	IIIa	yes	–
13	68	F	No	Full	65	80	IIIa	II	yes	–
16	68	F	No	Full	85	95	IIb	II	yes	–
17	74	F	Yes	Full	50	65	IIa	IIIa	yes	–
18	78	F	No	Full	70	90	IIIa	IIIa	yes	–
Mean	71.5				66.5	81.8				
S.D.	9.6				14.3	10.4				

Out of 20 included patients, one trochanteric fracture patient died during follow-up unrelated to the implantation of CERAMENT™|G (myocardial infarction), and one refused follow-up, leaving 8 trochanteric fracture and 10 revision of THA patients for final analysis.

Radiological analysis of trochanteric fractures showed that reposition of bone fragments was satisfactory according to previously described criteria in all cases. AP view evaluation revealed that the dynamic screw was placed in the center of the femoral head for 9 patients while for one patient it was placed inferiorly, while in lateral view all dynamic screws were placed centrally. Mean TAD value was 19.7 mm (±SD 3.3 mm, range 14.3–25.2 mm) (Table 3).

**Table 3:** Data of the trochanteric fractures - screw placement, angles and neck shortening

Patient No.	Fracture type	Screw position	TAD (mm)	CCD angle after op. (°)	CCD angle healthy leg (°)	CCD angle at 3 months (°)	CCD angle 1 year (°)	Neck shortening at 3 months (mm)	Total shortening at 1 year (mm)
1	A3.1	Central/Central	25.2	131	134	130	128	11.8	13.4
2	A1.2	Central/Inferior	14.3	133	130	–	–	–	–
3	A2.1	Central/ Central	22.2	130	128	127	127	4.7	6.5
4	A1.2	Central/ Central	17.8	129	130	129	128	2.4	2.7
5	A2.1	Central/ Central	16.2	120	130	died	–	–	–
8	A1.2	Central/ Central	19.7	134	131	133	133	2.0	2.2
14	A2.1	Central/ Central	17.6	132	127	128	128	0.7	1.1
15	A1.1	Central/ Central	18.6	129	130	126	126	0.8	0.9
19	A1.3	Central/ Central	20.3	132	126	130	130	1.0	1.2
20	A1.1	Central/ Central	24.6	129	125	129	–	1.2	
Mean			19.7	129.9	129.1	129.0	128.6	3.1	4.0
S.D.			3.3	3.7	2.5	2.0	2.1	3.5	4.2

Table shows the type of the fracture according to the AO classification, the placement of the lag screw in femoral head, tip-apex distance (TAD), caput-collum-diaphyseal (CCD) angles and femoral neck shortening after 3 months and 1 year follow-up.

The mean sliding distance of the screw (shortening of the femoral neck) was 3.1 mm (±SD 3.5 mm, range 0.7–11.8 mm) after 3 months and remained almost unchanged at 1 year follow-up (Table 3). Caput-collum-diaphyseal (CCD) angles of the operated hips were in the range of 120°-134° (mean 129.9° ± SD 3.7°), and similar to the CDD angles of the healthy contralateral hip (mean 129.1° ± SD 2.5°) (Table 3), with no varus or valgus deformity after fracture fixation. Radiological bone healing was observed in all available trochanteric fracture patients for follow-up.

At 3 months follow-up, 7 patients were walking independently, with no need of walking aids, while one patient had some walking problems and used a cane for support. The same results were observed at 1 year follow-up (Table 1).

Radiological visualision of CERAMENT™|G was possible on postoperative x-rays, however at 3 months and 1 year follow-up the material was difficult to differ from new bone growing into the apatite scaffold (Fig. 1).

**Fig. 3:**
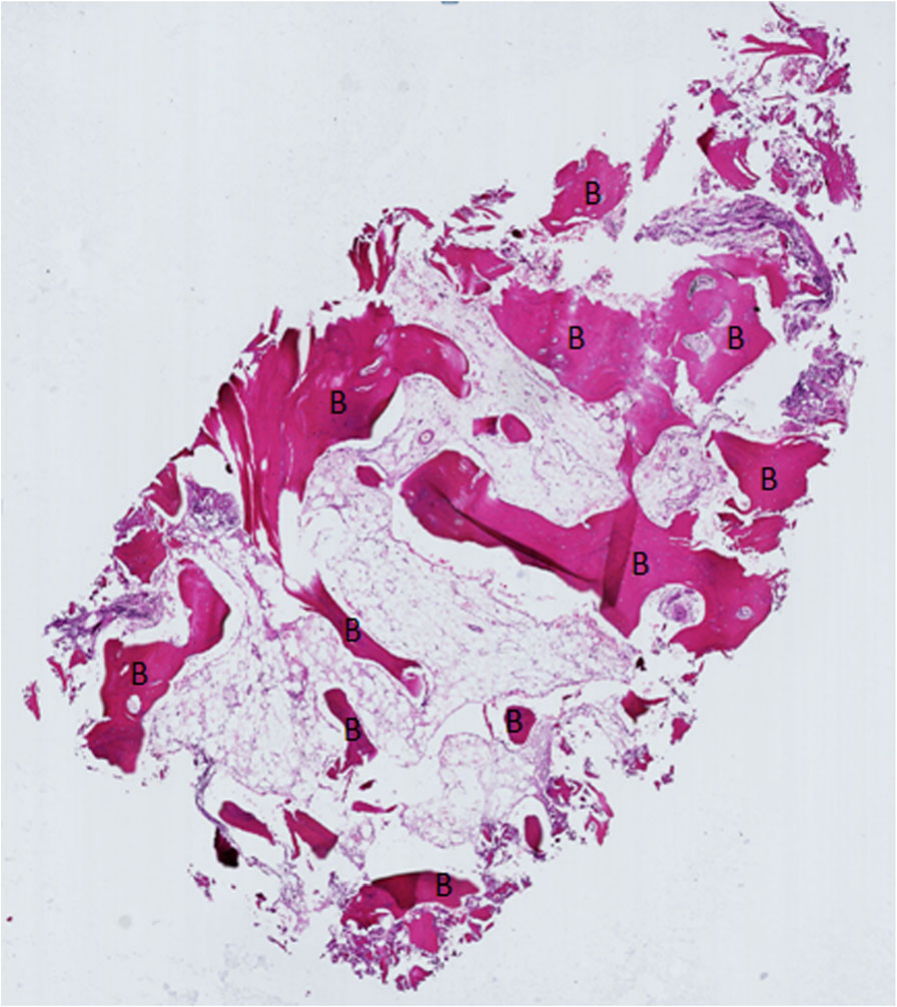
Specimen of the tissue surrounding the removed lag screw shows viable and mature bone (B)

**Fig. 4:**
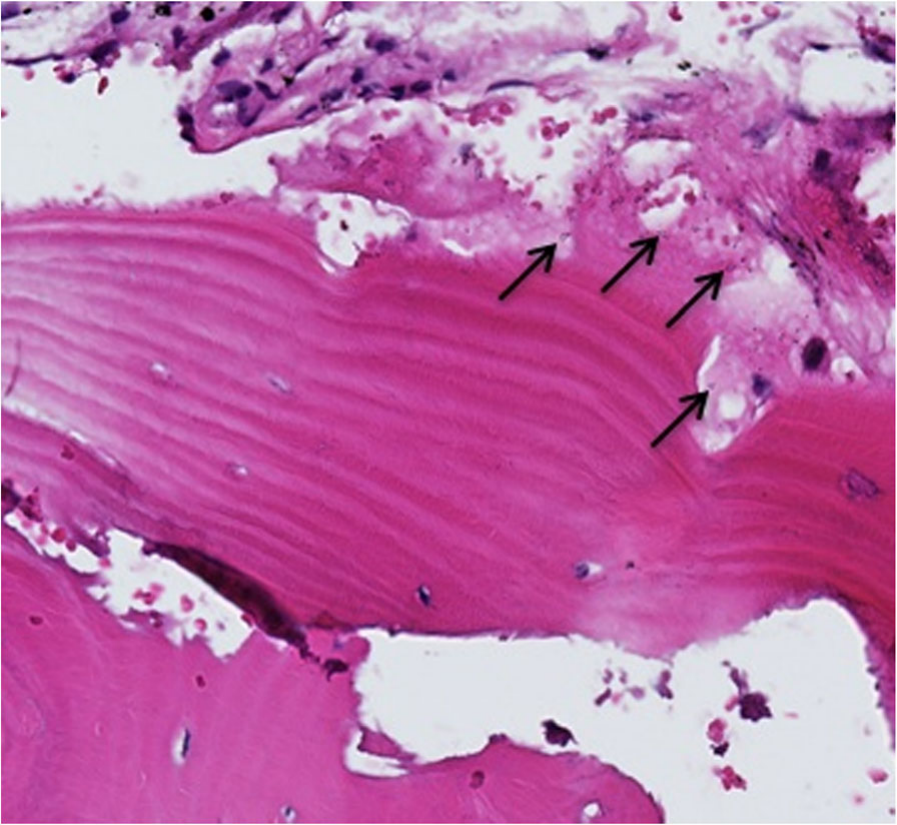
Higher magnification shows the presence of hydroxyapatite conglomerate (indicated by the black arrows)

**Fig. 5:**
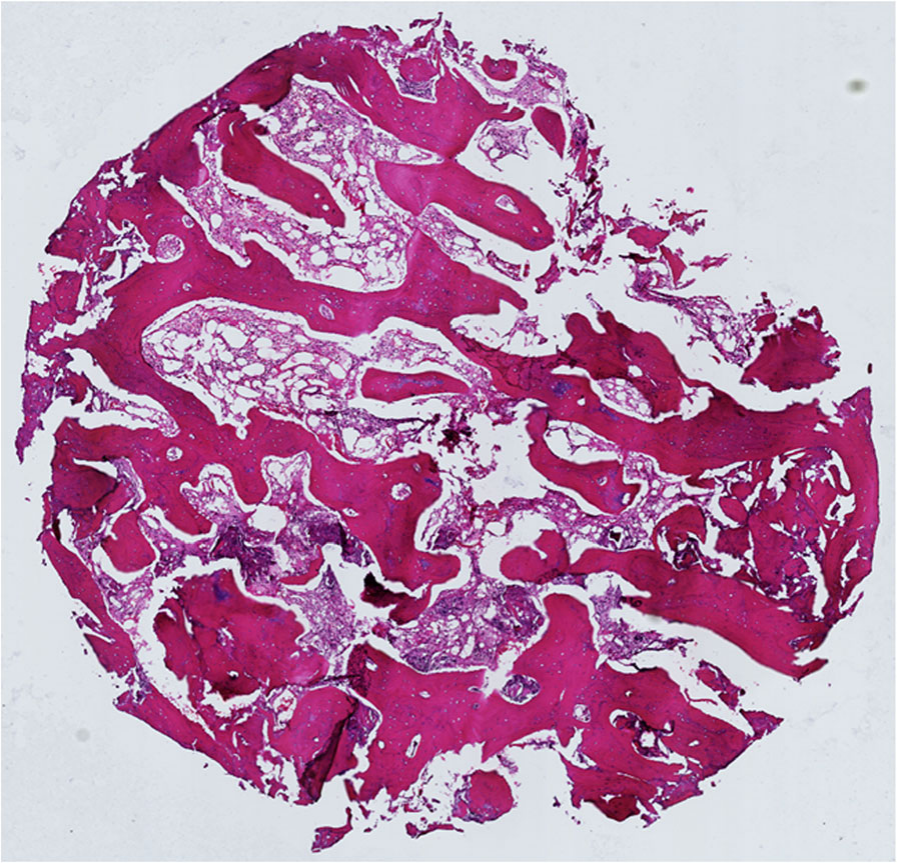
A second biopsy of the tissue surrounding the removed lag screw. Islands of viable trabecular bone is seen and the space between trabeculae is filled with marrow-like tissue

One trochanteric fracture patient (No 3) had additional surgery after 1 year follow-up, the hardware was removed due to some discomfort in trochanteric region (Fig. 1). During the surgery, the extraction of the dynamic screw was extremely difficult, probably due to excessive bone ongrowth on the hardware. Bone biopsies were taken from the trochanteric region for subsequent analysis. Histological analysis showed viable and mature bone in all biopsies (Fig. 3). High magnification of one of the specimens showed the presence of a loose hydroxyapatite conglomerate (indicated by the black arrows in Fig. 4). A second specimen showed islands of viable trabecular bone and the space between the trabeculae was filled with marrow like tissue (Fig. 5).

All 10 revision of THA patients reported having full recovery at 3 months, which was unchanged at 1 year follow-up. Two complained of light pain in the operated hip (Table 2) but radiological analysis showed that the bone density had increased in the proximal part of the femurs, where the bone defect was filled with CERAMENT™|G. No bone resorption was observed. No signs of osteolysis or loosening were observed in the acetabular or femoral components (Table 2).

Mean value of VAS for quality of life for the hip revision patients after 3 months was 66.5 (±SD 14.3, range 40–90) and at 1 year 81.8 (±SD 10.4, range 65–100), while for DHS patients the mean value was 71.3 (±SD 11.4, range 50–80) at 3 months and 84.8 (±SD 4.8, range 80–90) at 1 year follow-up (Tables 1 and 2).

None of the patients had impaired hearing, kidney function or any allergic reactions to gentamicin. No wound complications or local infections were registered in any patient.

## Discussion

To our knowledge, this is the first study investigating the local use of biodegradable bone graft substitute with antibiotics for bone defect reconstructions in aseptic revision of THA and trochanteric fracture patients.

A previous study showed that gentamicin from CERAMENT™|G elutes with a high burst rate through the first days after operation [7]. The local level of the concentration is sufficient not only to prevent infection, but also for eradication of recurrent bone or joint infections [11], thus is it not surprising that no signs of infection were observed in our investigated group.

The bone graft substitute does not improve immediate prosthesis-bone interface strength. In short term the use of a proximal bone graft substitute might not be necessary as a distally fixed stem would provide sufficient support. However, after remodeling, the proximal bone graft substitute gives mechanical support due to increased new bone formation around the prosthesis. The antibiotic elusion provided from the bone graft substitute protects the bone bed from infections. This is in concordance with findings reported in previously performed in vivo studies, where an uncemented knee joint prostheses and CERAMENT™|G were implanted in rabbits and showing better implant fixation in the host bone as compared to the control group without CERAMENT™|G [5]. Our experience of removing a dynamic hip screw from the trochanteric region where CERAMENT™|G had been implanted was that extremely high forces were needed. We assume that excessive new bone formation, probably related to CERAMENT™|G implantation and subsequent ongrowth into the sliding screw may explain the difficulties in hardware removal. However, no conclusions can be drawn from this limited case series and additional comparative studies need to be carried out. Histology confirmed new bone formation growth into the ceramic material.

Radiological analysis of the trochanteric fracture patients after DHS implantation showed a low level of dynamic screw migration, with an avarage of 3 mm at 3 months in our group with osteoprotic geriatric patients, while in a larger study by Platzer et al. a mean value of screw migration together with femoral neck shortening in non geriatric patients was reported to be 11 mm at 1 year follow-up [12]. Augmentation with a slow resorbing calcium phosphate (apatite) has not been shown to improve fixation in femoral neck fractures [10]. Since long term fixation is dependent on the amount and quality of new bone formed around the implant, we believe that the use of a biphasic material with part being replaced by ingrowing bone that surrounds the apatite may be beneficial for fracture stability and implant fixation. By adding antibiotics to the bone graft substitute, it is in addition possible to prevent surgical site infections in surgery carrying high risk [11]. The disadvantage of using antibiotics as standard in ceramic material may lead to over-use and not be necessary for all patients and even be harmful for patients suffering from renal insufficiency [13]. We suggest that CERAMENT™|G implantation at the fracture site could improve healing, bone formation and stabilization in the trochanteric fractures, thus preventing dynamic screw migration and shortening of the femoral neck.

There are several limitations in this study. The number of patients included is low and the study should be considered as a clinical feasibility study to show for the first time clinical outcome of an antibiotic eluting injectable bone substitute CERAMENT™|G used in major hip surgery. The prime objective was to extensively analyze the pharmacokinetics which has been reported elsewhere [7], but it contained no clinical follow-up. The study reports trochanteric fractures and revision THA, which are different conditions and with different outcome. The methods for clinical follow-up could have used in addition to VAS other patient related outcome instruments.

## Conclusions

Our limited study indicates that local CERAMENT™|G could be safely used in revision of THA and trochanteric fractures, to prevent infection and fill bone defects. No local or systemic complications were found and circulation disturbances in the femoral head were not seen.

## Abbreviations

AP: Anteroposterior
CCD: Caput-collum-diaphyseal
DHS: Dynamic hip screw
EDTA: Ethylenediaminetetraacetic acid
H&E: Haematoxylin and eosin stain
PMMA: Polymethylmethacrylate
PPS®: Porous Plasma Spray
QoL: Quality of Life
TAD: Tip-apex distance
THA: Total hip arthroplasty
VAS: Visual Analog Scale
